# Immune-enhancing effect of nano-DNA vaccine encoding a gene of the prME protein of Japanese encephalitis virus and BALB/c mouse granulocyte-macrophage colony-stimulating factor

**DOI:** 10.3892/mmr.2015.3419

**Published:** 2015-03-04

**Authors:** YONGZHEN ZHAI, YAN ZHOU, XIMEI LI, GUOHE FENG

**Affiliations:** Department of Infectious Diseases, Shengjing Hospital of China Medical University, Shenyang, Liaoning 110004, P.R. China

**Keywords:** Japanese encephalitis virus, chitosan, granulocyte-macrophage colony-stimulating factor, dendritic cells, nanoparticles, cytotoxic T-lymphocytes

## Abstract

Plasmid-encoded granulocyte-macrophage colony-stimulating factor (GM-CSF) is an adjuvant for genetic vaccines; however, how GM-CSF enhances immunogenicity remains to be elucidated. In the present study, it was demonstrated that injection of a plasmid encoding the premembrane (prM) and envelope (E) protein of Japanese encephalitis virus and mouse GM-CSF (pJME/GM-CSF) into mouse muscle recruited large and multifocal conglomerates of macrophages and granulocytes, predominantly neutrophils. During the peak of the infiltration, an appreciable number of immature dendritic cells (DCs) appeared, although no T and B-cells was detected. pJME/GM-CSF increased the number of splenic DCs and the expression of major histocompatibility complex class II (MHCII) on splenic DC, and enhanced the antigenic capture, processing and presentation functions of splenic DCs, and the cell-mediated immunity induced by the vaccine. These findings suggested that the immune-enhancing effect by pJME/GM-CSF was associated with infiltrate size and the appearance of integrin αx (CD11c)+cells. Chitosan-pJME/GM-CSF nanoparticles, prepared by coacervation via intramuscular injection, outperformed standard pJME/GM-CSF administrations in DC recruitment, antigen processing and presentation, and vaccine enhancement. This revealed that muscular injection of chitosan-pJME/GM-CSF nanoparticles may enhance the immunoadjuvant properties of GM-CSF.

## Introduction

Japanese encephalitis (JE) is a mosquito-borne viral disease causing infection of the central nervous system in southeastern and far eastern Asia (1). Every year, more than 50,000 cases are reported, with a patient mortality rate of between 25 and 30%, while 50% are left with permanent central nervous system sequelae (2). Vaccination has been observed to protect against Japanese encephalitis virus (JEV) infection in humans and domestic animals (3–5). A mouse brain-grown formalin-inactivated JEV vaccine is available internationally, however this vaccine has several limitations, including the high cost of production, lack of long term immunity and allergy risk due to the presence of murine encephalogenic basic proteins or gelatin stabilizer (6–9). Therefore, there is a requirement for a safer, more effective and cheaper vaccine to be developed for protection against JEV infection. In recent years, a marked improvement has been made in plasmid DNA-based vaccinations, in order to overcome the disadvantages of traditional JEV vaccines.

Plasmid DNA-based vaccination strategies have become investigated extensively over the past decade due to their potential to fulfil the requirement of safer and cheaper vaccines (10). A number of candidate DNA vaccines against JEV have been developed using plasmids, which express various structural or non-structural JEV proteins and, in a mouse model, these plasmids have been found to provide different degrees of protection against challenge with a lethal dose of JEV (11). Plasmids expressing the JEV envelope (E) protein are the most promising as they induce JEV neutralizing antibodies, which are important indicators of protectin (12). A plasmid DNA encoding premembrane (prM) and emvelope (E) proteins may provide a more effective DNA vaccine compared with a construct expressing E protein alone (13,14). However, as with other DNA vaccines, the immune effect of plasmid DNA encoding of JEV prM and E proteins remains lower compared with that of an inactivated vaccine, purified from infected adult mouse brain (15,16). Therefore, enhancing the immunogenicity of DNA vaccines has become the key in their investigation and development.

Following DNA vaccination, the limited number of DCs at the injection site can uptake plasmid DNA or antigens (Ag) expressed by muscular cells that have been transfected with plasmid DNA, and activate the cell-mediated and humoral immune responses through different Ag-presenting pathways. The quantity of DC recruitment at the injection site during Ag expression is a possible rate-limiting factor for the effectiveness of DNA vaccines (17). Use of a combination of immunomodulatory adjuvants, which code for maturation, activation or recruitment factors, aims to enable further manipulation of antigen-presenting cells (APC) *in vivo* to enhance the potency of DNA vaccines.

Granulocyte-macrophage colony-stimulating factor (GM-CSF) is an important cytokine for the generation of myeloid-associated DCs *in vitro*, however, the role of this cytokine *in vivo* remains to be elucidated. Certain studies have revealed that a polyethylene glycol modified form of GM-CSF (pGM-CSF) resulted in a marked expansion of DC numbers *in vivo* (18,19). Additionally, other studies have indicated that GM-CSF is able to modulate immune responses *in vivo*. Transplantation of tumors transduced with GM-CSF results in the expansion of DCs *in vivo* (20,21) and the generation of antitumor immune responses (22,23). Similarly, administration of GM-CSF enhances DC recruitment, antigen presentation and a vaccine-induced immune response (24).

Non-viral delivery systems for gene therapy have been increasingly advocated as safer alternatives to viral vectors (25,26). Cationic polymers have been revealed as a promising carrier among the non-viral gene delivery systems (27). Due to its cationic nature, chitosan has been widely assessed as a non-viral gene delivery system and has been successfully used as a nasal delivery system for subunit influenza vaccine (28), tetanus toxoid (29) and diphtheria (30). However, the effect of chitosan as an adjuvant via muscular injection remains to be fully elucidated.

In the present study, the encoding genes for mouse GM-CSF, JEV prM and E proteins were cloned into the same eukaryotic expression vector to construct a fusion plasmid, termed pJME/GM-CSF. The chitosan-pJME/GM-CSF nanoparticles were prepared and BALB/c mice were vaccinated with the pJME/GM-CSF plasmid and the prepared chitosan-pJME/GM-CSF nanoparticles via intramuscular injection. The kinesis of cell infiltration at the injection site was observed and the number, phenotype and function of splenic DC, as well as the induced cell-mediated immune response, were assayed. The immune-enhancing effect and mechanism induced by the GM-CSF-encoded gene and the adjuvant effect of chitosan via intramuscular injection were investigated.

## Materials and methods

### Preparation and identification of chitosan-pJME/GM-CSF nanoparticles

The recombinant plasmid, termed pJME, encoding the JEV prME protein, and the pJME/GM-CSF encoding fusion protein of prME and GM-CSF were developed and stored in the Laboratory of Infectious Diseases, Shengjing Hospital of China Medical University (Shenyang, China). The chitosan-pJME/GM-CSF nanoparticles were prepared, as described previously (31). In brief, 100 *μ*l chitosan (Sigma-Aldrich, St. Louis, MO, USA) at 0.02%, w/v in 5 mM NaAc-Hac buffer (pH 5.5) was added, during high-speed vortexing (G560E; Scientific Industries, Inc., Bohemia, NY, USA), to 100 *μ*l plasmid DNA (100 *μ*g/ml in 50 mM Na_2_SO_4_) for 1 min. The two solutions were preheated to 50–55°C separately. The formulations were then lyophilized for 24 h with either 1% sucrose, 1% trehalose, or no cytoprotectant. Lyophilization was carried out after adding the suitable cytoprotectants (1% w/v mannitol) to the formulations in glass ampoules for 24 h at 1 mbar pressure and −110°C (Maxi Dry; Jouan Nordic A/S, Allerød, Denmark), in order to obtain free-flowing powder. The prepared nanoparticles were maintained in a dessicator (S250-II; Shreyans Industries Ltd., New Delhi, India) at 2–8°C until required. Prior to administration into mice, the lyophilized DNA formulation was reconstituted with ultrapure water.

Gel retardation analysis: The chitosan-pJME/GM-CSF nanoparticles were obtained and analyzed in a 0.8% agarose gel containing 1 *μ*g/ml ethidium bromide, in Tris-borate-EDTA (TBE) buffer. The pJME/GM-CSF were visualized under UV light using a gel documentation system (Biospectrum HR410; UVP, LLC, Upland, CA, USA).

Entrapment efficiency is the percentage of containment of pJME/GM-CSF by chitosan (30). The prepared nanoparticles were centrifuged at high speed at 4°C (12,000 × g for 1 h). A UV spectrophotometer (UV-2450; Shimadzu Corporation, Kyoto, Japan) was used to measure the concentration of pJME/GM-CSF in the supernatant. The entrapment efficiency was calculated using the following formula: Entrapment efficiency (%) = (WTotal − WFree) / WTotal × 100%, where WTotal was the total quantity of pJME/GM-CSF added and WFree was the quantity of pJME/GM-CSF in the supernatant.

Characterization of chitosan-DNA nanoparticles: A small quantity (0.5 ml) of the prepared nanoparticles were obtained and placed on a copper net with a carbon membrane for 2 min. Filter paper was used to dry the liquid and 2% phosphotungstic acid was then added for 2 min for staining. Morphological examination of the nanoparticles was observed and images were captured using transmission electron microscopy (LIBRA 120; Carl Zeiss, Thornwood, NY, USA). In addition, an adequate quantity (10 ml) of prepared nanoparticles were acquired and diluted with double-distilled water (1:1), prior to measurement of the average diameter and zeta-potential using a nanoparticle analyzer (Zetasizer3000HS Malvern Instruments, Southborough, MA, USA).

Protective effect of nanoparticles to the plasmid: The chitosan-pJME/GM-CSF nanoparticles were incubated with 5 U/*μ*l DNAse I (Gibco-BRL, Carlsbad, CA, USA) for 15 min at 37°C. The reaction was terminated by adding iodoacetic acid (DNAse I inhibitor) solution to a final concentration of 5 mM. The samples were analyzed on a 0.8% agarose gel containing 1 *μ*g/ml ethidium bromide in TBE buffer. The pJME/GM-CSF was visualized under UV light using a gel documentation system.

### Animals and immunization procedure

Female, four-week-old BALB/c mice (weighing 14–16 g) were obtained from the Institute of Laboratory Animal Sciences, Chinese Academy of Medical Sciences (Beijing, China) and maintained in sterile cages under specific pathogen-free conditions. The mice were provided *ad libitum* access to normal chow and water, and were maintained under a normal diurnal cycle at room temperature (22°C). The mice were divided into six groups (n=33/group), as follows: Chitosan-pJME/GM-CSF nanoparticles; pJME/GM-CSF; pJME; JE-inactivated vaccine as a positive control; chitosan solution and pcDNA3.1 (+) as negative controls. The plasmids were administered in a total volume of 50 *μ*l into the quadriceps muscle mass on the left hind leg of the mice in each group at the following concentrations: Chitosan-pJME/GM-CSF nanoparticles with 100 *μ*g pJME/GM-CSF; phosphate-buffered saline (PBS) with 100 *μ*g pJME/GM-CSF; PBS with 100 *μ*g pJME; JE-inactivated vaccine as the positive control, chitosan solution and PBS with 100 *μ*g pcDNA3.1(+) as negative controls. Mice received their first immunization between 4 and 6 weeks of age. At 3 and 5 weeks following primary injection, the mice received two booster doses in the same muscle, containing the same quantity of plasmid as in the primary dose. The positive control group comprised mice immunized with an inactivated vaccine, which comprised a formalin-inactivated mouse brain-derived JEV vaccine (Beijing-1 strain) obtained from the Liaoning Province Center of Disease Control and Prevention (Shenyang, China). Each mouse in this inactivated-vaccine group was injected with 100 *μ*l (1/5 of the recommended adult dose) inactivated vaccine. At different time-points, the mice were sacrificed by cervical dislocation following anesthesia with 10% chloral hydrate (Qingdao Yulong Seaweed Co., Ltd, Qingdao, China). The present study was approved by the Ethics Committee of Shengjing Hospital of China Medical University. The present study was performed in strict accordance with the recommendations of the Guide for the Care and Use of Laboratory Animals of the National Institutes of Health. The animal use protocol was reviewed and approved by the Institutional Animal Care and Use Committee of China Medical University.

### Histology and immunohistochemistry

For histological and immunohistochemical analyses, the injected muscles were removed from two mice daily for 7 days and on day 14 following injection. Standard hematoxylin and eosin (H&E; Sigma-Aldrich) staining was performed to assess cell infiltration and inflammatory infiltrate in the injected muscles. The injected muscle tissues were removed, fixed in 10% formalin [Sigma-Aldrich (Shanghai) Trading Co., Ltd., Shanghai, China], embedded in paraffin [Sigma-Aldrich (Shanghai) Trading Co., Ltd.], and sectioned (6 *μ*m) for histological analysis. Staining and grading of the cell infiltrates was performed, as described previously (32): 0=no infiltrate; 1+=one small cell cluster; 2+=two small or moderate size cell clusters and 3+=extensive, multifocal cell infiltration.

To identify the types of infiltrative cells recruited to the injection site, the muscle sections were analyzed by immunohistochemistry. Muscles were snap frozen by overlaying with Histo-Prep tissue-embedding medium (Thermo Fisher Scientific, Waltham, MA, USA) and immersing in liquid nitrogen-cooled isopentane for 20 sec. All the samples were stored at −70°C until analysis. Serial frozen sections (6 *μ*m thickness) from each muscle were adhered to Superfrost Plus slides (Thermo Fisher Scientific), fixed in ice-cold acetone [Sigma-Aldrich (Shanghai) Trading Co., Ltd.] at −20°C for 10 min, air-dried and rinsed in distilled water to remove embedding medium. The muscle sections were mounted on glass slides (Thermo Fisher Scientific) and the slides were washed in PBS three times for 5 min each, incubated in 0.2% Triton-X100 in PBS for 15 min and washed again in PBS. The samples were incubated with 1% bovine serum albumin (Sigma-Aldrich) in PBS (PBS-BSA) for 30 min at room temperature to inhibit nonspecific binding. The sections were then incubated for 2 h at 37°C with primary antibodies (Abs), rat monoclonal anti-Mac-3 (M3/84; cat. no. 550292; 1:50), integrin αx chain (CD11c; HL3; cat. no. 550283; 1:50), IAd/Ed (2G9; cat. no. 556999; 1:50) and GR-1 (Ly-6 G; cat. no. 550291; 1:50), all from BD Pharmingen, San Diego, CA, USA, according to the manufacturer’s instructions, followed by 1 h incubation with 5 *μ*g/ml biotinylated rabbit anti-rat secondary Ab (sc-358919; 1:400; Santa Cruz Biotechnology, Santa Cruz, CA, USA) at 37°C. Following 30 min incubation with streptavidin-peroxidase (BD Pharmingen) at 37°C, Ag-Ab reactions were developed using 2mg/ml 3,3′-diaminobenzidine (Dako, Carpinteria, CA, USA) as the substrate. The slides were washed twice with PBS between each incubation step. All reagents were added in a volume of 50 *μ*l, and the incubations were performed at room temperature in a humidified chamber. Staining and grading of the cell infiltrates were performed, as described previously (31): 0=no cells stained, 1+=fewer than 10% cells stained, 2+=10–50% stained and 3+=50–100% cells stained.

### Measurement of splenic DCs

Single-cell suspensions from control and experimental spleens were prepared by cutting the spleen into small pieces and then performing digometry following incubation of the cells with fluorescein isothiocyanate (FITC)-CD11c monoclonal antibody (MAb; BD Pharmingen) for 30 min at 4°C. The prepared single cell suspensions were separated into low and high density fractions on a Percoll gradient (Sigma-Aldrich; P=1.077), with the low density fraction being the enriched DC. Phenotyping of the spleen-derived DCs was performed by incubating the DC-enriched cells with phycoerythrin (PE)-labeled anti-CD11c, FITC-labeled anti-cluster of differentiation (CD)80 (B7-1) MAb, FITC-labeled anti-CD86 (B7-2) MAb, and FITC-labeled anti-major histocompatibility complex class II (MHCII; BD Pharmingen) on ice for 30 min, followed by washing with PBS. Rat PE-immunoglobulin (Ig)G and FITC-IgG were used as isotype controls. A total of 10,000 cells were collected for each sample and the data were analyzed using CellQuest software, version 5.1 (BD Biosciences, San Diego, CA, USA). The results were expressed as the percentage of positive cells.

### Measurement of Ag uptake

Following incubation with FITC-dextran (Molecular Probes, Eugene, Oregon, USA), at 37°C for 0, 5, 15, 30, 45 and 60 min, the enriched DCs were washed three times in PBS-5% fetal bovine serum (FBS; Gibco-BRL). The FITC-dextran uptake was quantified as the mean fluorescence intensity (MFI). Nonspecific FITC signal was assessed by incubating the cells in FITC-dextran, at 0°C. All incubations were performed in PBS-5% FBS. To verify that the flow cytometry-based FITC signal was representative of internalized dextran, the cells were analyzed by epifluorescence and phase-contrast microscopy. Epifluorescence and phase-contrast microscopy were performed using a microscope (DM IRBE; Leica Microsystems, Wetzlar, Germany) equipped with an Orca model C4742-5 charge-coupled device (Hamamatsu Photonics, Hamamatsu, Japan).

### Processing of ovalbumin (OVA) into peptide

The cells were pulsed with DQ-olvalbumin (OVA;Molecular Probes) for 15 min at 37°C, and then washed extensively with PBS, 5%FBS at 4°C. The cells were transferred to 37°C and processing of OVA into peptide was assayed by increasing the MFI over time. The DQ-OVA was quantified by flow cytometry, as in the Ag capture assays mentioned above. The DQ-conjugated OVA peptide was quantified using the FITC channel of a FACSCalibur (BD Biosciences).

### Mixed-lymphocyte reaction (MLR)

The ability of DCs to stimulate T lymphocyte proliferation is a hallmark of DC maturation, which reflects the antigen-presenting capacity of DCs. Single-cell suspensions in the spleen were prepared, as described above, which were separated into low and high density fractions on a Percoll gradient (P=1.077). The low density cells were placed in a culture bottle, and incubated at 37°C for 3 h in RPMI 1640 (Gibco-BRL) medium containing 10% FBS. The low density cells were cultured for a further 18 h following removal of the non-adherent cells. The adherent cells were transferred to a 24-well culture plate coated with human serum IgG, and non-adherent cells were collected after 1 h. The DCs prepared using this method were at least 80% pure. The cells were cultured for 36 h in the presence of 20 ng/ml rGM-CSF and interleukin (IL)-4 (Abcam, Burlingame, CA, USA) and were used as the stimulator cells. Responder T lymphocytes were derived from the spleen of isogeneic BALB/c mice using a lymphocyte separation column (Cedarlane, Grand Island, NY, USA). MLR assays were performed in 96-well, round-bottomed culture plates (Falcon; BD Biosciences) in 2 ml complete medium, including 1/20 volume of live JEV (Beijing-1 strain; 10^3^ PFU/ml). Isogeneic T cells (2×10^5^) were incubated with DC-enriched spleen cells (5×10^3^) treated with mitomycin (25 *μ*g/ml; Sigma-Aldrich). The cells were cultured in 0.2 ml RPMI 1640 containing 10% FCS in a humidified CO_2_ incubator for 3 days. After culturing for 72 h, 10 *μ*l MTT (5 g/l; Beyotime Institute of Biotechnology, Nantong, China) was added to the wells, and the plates were incubated for 6 h. Subsequently, 150 *μ*l/well dimethyl sulfoxide (Sigma-Aldrich) was added, and the absorbance was measured at 570 mm using a spectrophotometer reader (UV-2450). Each well was measured three times, and each sample was assayed in triplicate. Untreated cells cultured in medium alone were used as controls.

### Cytotoxic T cell lysis (CTL) assay

Cytotoxicity assays examining the release of lactate dehydrogenase (LDH) activity were performed on week 8 (3 weeks after the final DNA inoculation), as previously described (33). P815 cells were purchased from Shanghai Institutes for Biological Sciences (Shanghai, China) and the JEV-infected P815 cells were used as target cells. The effector cells were spleen cells isolated from the BALB/c mice in each experimental group. In a typical procedure, the target cells were distributed into quadruplicate wells of a 96-well plate (5×10^3^ cells/well) and the effector-to-target cell ratio was adjusted to 10:1, and incubated in a humidified chamber at 37°C and 5% CO_2_ for 5 h prior to collection of the supernatant. The LDH activity released into the supernatant was measured using a Cytotox 96 assay kit (Promega Corporation, Madison, WI, USA), according to the manufacturer’s instructions. The percentage of specific lysis was calculated as follows: (experimental LDH release − spontaneous LDH release) / (maximum LDH release − spontaneous LDH release) × 100.

### Statistical analysis

All the values are expressed as the mean ± standard deviation. Statistical analyses of the experimental data and controls were performed by one-way factorial analysis of variance. All statistical analyses were conducted using SPSS 17.0 (SPSS Inc., Chicago, IL, USA). P<0.05 was considered to indicate a statistically significant difference.

## Results

### Identification of chitosan-DNA nanoparticles

**Figure 1:**
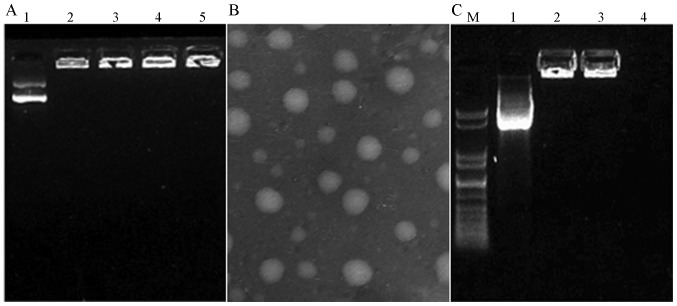
Identification of chitosan-pJME/GM-CSF nanoparticles. (A) Agarose gel electrophoresis of chitosan-pJME/GM-CSF nanoparticles. Lane 1, pJME/GM-CSF; lane 2, 3 and 4, chitosan-pJME/GM-CSF nanoparticles. (B) Transmission electronic micrograph of chitosan-pJME/GM-CSF nanoparticles (magnification, ×50,000). (C) Protection of chitosan-pJME/GM-CSF nanoparticles. M, DNA Marker; lane 1, pJME/GM-CSF; lane 2, chitosan-pJME/GM-CSF nanoparticles +DNAse I; lane 3, chitosan-pJME/GM-CSF nanoparticles; lane 4, pJME/GM-CSF+DNAse I. pJME, premembrane and envelope proteins derived from Japanese encephalitis virus; GM-CSF, granulocyte-macrophage colony-stimulating factor.

Gel retardation analysis revealed that pJME/GM-CSF was almost completely retarded in the loading well, suggesting that the majority of pJME/GM-CSF bound with chitosan, and the entrapment efficiency of chitosan-pJME/GM-CSF nanoparticle was 92% (Fig. 1A).

Visualization of the chitosan-pJME/GM-CSF nanoparticles by transmission electronic microscopy found the nanoparticles to be almost spherical in shape and ~50–150 nm in size. The average diameter of the prepared nanoparticles, determined using a Zetasizer was found to be 108.3 nm (Fig. 1B), and the Zeta potential of the prepared nanoparticles was 10.8±1.3 mV at the complexation pH (pH 5.5) and 2.9±0.6 mV at pH 7.

The gel electrophoresis results revealed that chitosan effectively protected pJME/GM-CSF from degradation in an incubation duration of up to 15 min, whereas the DNA marker was completely degraded after 15 min (Fig. 1C).

### Kinetics and type of cell infiltration

**Figure 2:**
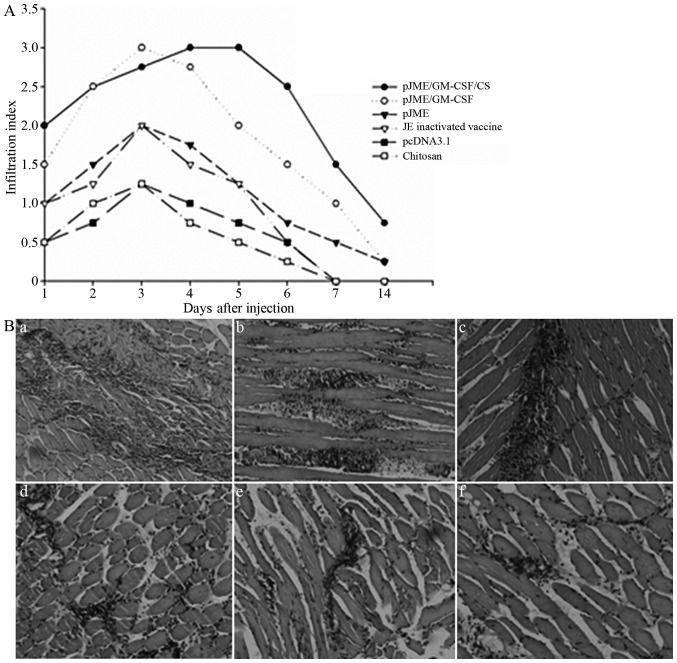
(A) Kinetics of cell infiltration in muscle and hematoxylin and eosin (H&E)-stained muscle sections 3 days after injection with various immunogens (magnification, ×100). Infiltration index was determined in H&E-stained muscle sections graded on a scale from 0–3. Infiltration index values represent the average of four muscles (standard deviation, 0–0.6). (B) Cell infiltration after intramuscular injection with various immunogens. Mice were injected with indicated immunogens in each gastrocnemius muscle, and both muscles were removed 3 days later. Frozen muscle sections were stained with H&E (magnification, ×100). (Ba) Chitosan-pJME/GM-CSF nanoparticles vaccinated group; (Bb) pJME/GM-CSF vaccinated group; (Bc) pJME vaccinated group; (Bd) JE inactivated vaccine group; (Be) pcDNA3.1(+) vaccinated group; (Bf) chitosan solution vaccinated group. GM-CSF, granulocyte-macrophage colony-stimulating factor; CS, chitosan; JE, Japanese encephalitis.

The results from H&E-stained muscle sections are presented in Fig. 2. The data are presented as the average infiltration grade of four muscles at each time-point. Chitosan solution and pcDNA3.1(+) led to small infiltrates, which persisted for 7 days. A significantly larger infiltrate was observed following injection of the pJME and JE-inactivated vaccines. pJME/GM-CSF produced infiltrates, which were significantly larger in duration and size compared with the pJME and JE-inactivated vaccines. This infiltrate, which reduced by day 7, reached its largest size at days 3, 4, and 5. The types and quantities of infiltration cells recruited by the chitosan-pJME/GM-CSF nanoparticles were similar to that of pJME/GM-CSF, and the duration of the peak of cell infiltration was longer, which appeared between day 3 and day 7 following immunization. In the majority of cases, one foci of inflammation was identified within the muscle.

**Figure 3:**
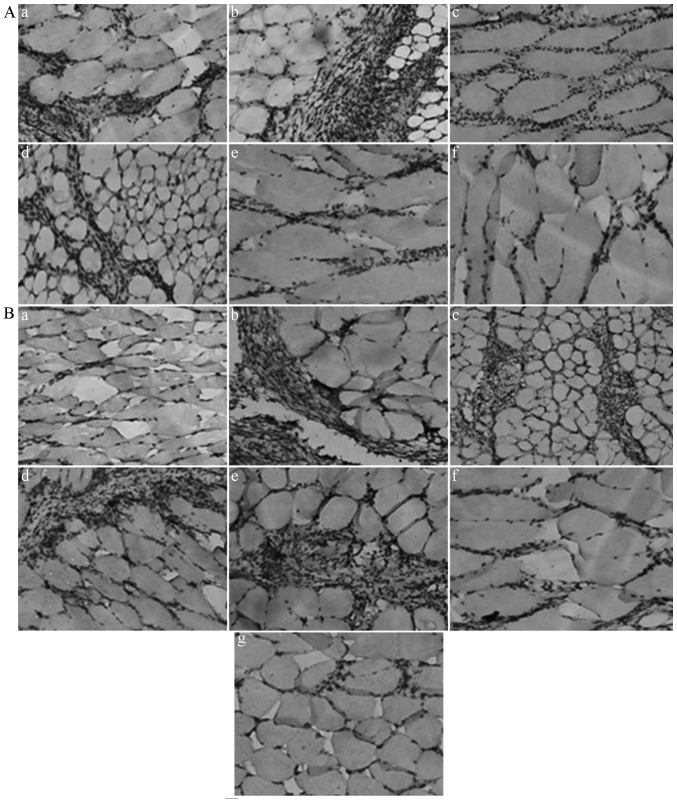
Effects of chitosan and GM-CSF-encoding gene are to participate in the recruitment of CD11c and increase infiltrate size and duration. Histological characterization of CD11c production in muscles of immunized mice. (A) CD11c-stained muscle sections 3 days after injection of various immunogens. (a) Chitosan-pJME/GM-CSF nanoparticles vaccinated group; (b) pJME/GM-CSF vaccinated group; (c) pJME vaccinated group; (d) JE inactivated vaccine group; (e) pcDNA3.1(+) vaccinated group; (f) chitosan solution vaccinated group (magnification, ×100). (B) CD11c-stained muscle sections after (a) 1, (b) 2, (c) 4,(d) 5, (e) 6, (f) 7 and (g) 14 days in the chitosan-pJME/GM-CSF nanoparticles-vaccinated group (magnification, ×100). CD11c; integrin, αx; pJME, premembrane and envelope proteins derived from Japanese encephalitis virus; GM-CSF, granulocyte-macrophage colony-stimulating factor.

In order to identify the type of infiltrating cells in the intramuscular injection site, the present study analyzed the type of infiltrating cells by immunohistochemical analysis using specific antibodies of different cell markers. The results from the muscles analyzed by immunohistochemistry 3 days after injection are presented in Table I. The predominant cell types found at the injection sites of each group were a population with a macrophage phenotype, which had marked staining with Abs to Mac-3 and I−A^d^/I−E^d^. These stained cells were observed throughout the entire 14 days of the study. Appreciable numbers of granulocytes, detected by Abs specific to GR-1, were also present in the infiltrated muscles. H&E staining revealed that these cells were predominantly neutrophils, with a small number of acidophils. Appreciable numbers of CD11c+ cells, the marker of DCs, were also detected in the infiltrates of the pJME and pJME/GM-CSF groups. No muscle sections were observed to present any reactivity with B7-1-specific Abs, which are a marker of APC activation and DC maturation. These CD11c+ cells were only detected 3, 4 and 5 days after immunization, which appeared later and disappeared earlier compared with the Mac-3+, IAd/Ed+ or GR-1+ cells. The types and quantities of cells in the infiltrate, recruited by the chitosan-pJME/GM-CSF nanoparticles, were similar to that by pJME/GM-CSF, whereas the duration of the peak of cell infiltration was longer, which was observed between day 3 and 7 after immunization; Fig. 3). No CD4+, CD8+T-lymphocytes or CD45+B-lymphocytes were detected, while the slides of normal spleen cells exhibited marked positive staining for antibodies of CD4, CD8, B220 and B7-1. No reactivity was observed with the isotype control primary Abs in any muscle.

### Concentration of CD11c+DC and MHC II expression

**Figure 4:**
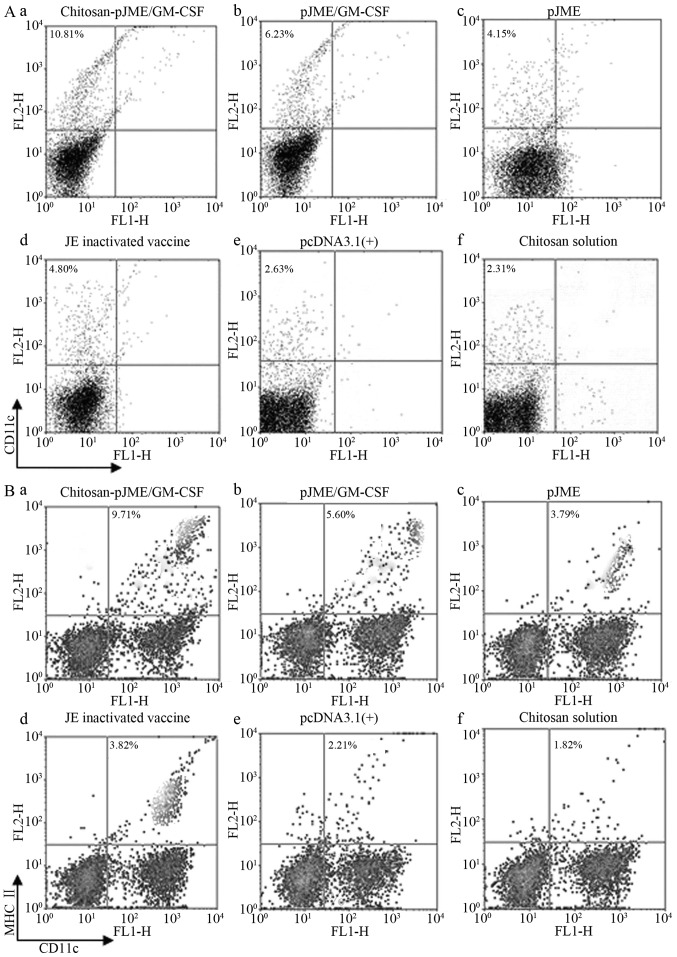
Effect of the chitosan and GM-CSF on surface expression of CD11c and MHC II on spleen dendritic cells from various vaccinated groups. (A) Spleen-derived DCs were incubated with antibodies targeting CD11c-FITC. Ther percentage of CD11c positive cells is indicated in the upper left quadrant of each panel. (B) Spleen-derived DCs were incubated with antibodies targeting CD11c-PE/MHCII-FITC. CD11c-PE/MHCII positive cells were assessed by flow cytometry. The percentage of double-positive cells is indicated in the upper right quadrant of each panel. Data represent four experiments (representative results denote the mean value of several individual mice. MHCII, major histocompatibility complex class II; CD11c; integrin, αx; GM-CSF, granulocyte-macrophage colony-stimulating factor; JE, Japanese encephalitis; DC, dendritic cell; FITC, fluorescein isothiocyanate; PE, phycoerythrin.

The adjuvant effects of chitosan and GM-CSF DNA on the CD11c+ DCs from the spleen were compared. The percentage of CD11c+ DCs in the spleen cells was 10.81% in the chitosan-pJME/GM-CSF nanoparticle-vaccinated group, which significantly increased the relative frequency of CD11c+ DCs subpopulations compared with the other groups (P<0.05). The percentage of CD11c+ DCs in the chitosan and pcDNA3.1(+)-vaccinated groups was 2.31 and 2.63%, respectively, and was, therefore, lower compared with all the other groups (P<0.05). The percentage of CD11c+ DCs in the spleen cells in the pJME/GM-CSF-vaccinated groups was higher compared with those in the pJME and JE-inactivated vaccine groups (P<0.05), however, no significant difference were observed in the levels of CD11c+ DCs between the pJME and JE-inactivated vaccine groups (P>0.05; Fig. 4A).

MHC and costimulatory molecules are necessary for Ag presentation by DCs, and the expression of MHC II expression is used to quantify the number of antigen cells (DCs, B cells and monocyte/macrophages) (26). The splenic CD11c+DCs from all the mice expressed negligible levels of CD80 and CD86, and high levels of MHC II; pJME/GM-CSF significantly increased the expression of MHC II in CD11c+DC compared with the pJME and JE-inactivated vaccine (P<0.05); Chitosan-pJME/GM-CSF nanoparticles significantly increased the expression of MHC II in the CD11c+DC compared with the pJME/GM-CSF-vaccinated group (P<0.05; Fig. 4B).

### Ag uptake

**Figure 5:**
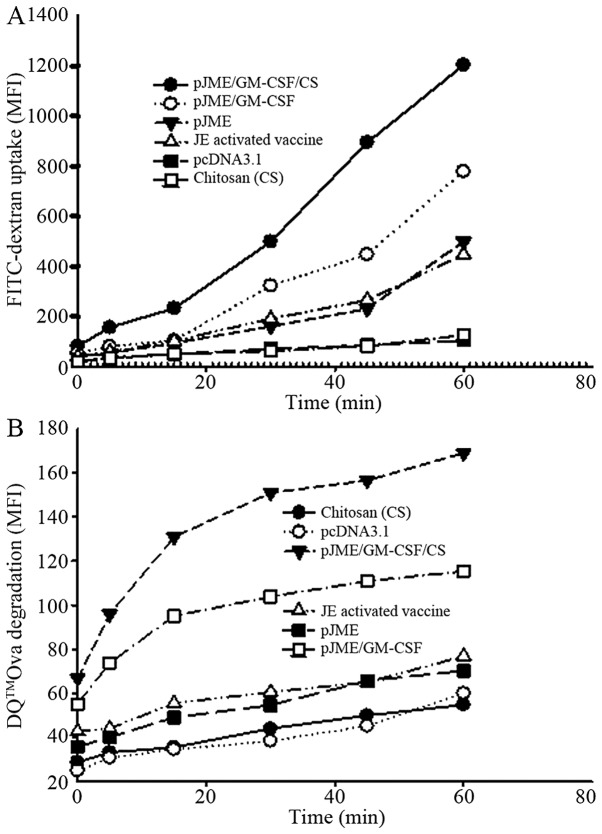
DCs from pJME/GM-CSF/CS-treated mice capture and process antigens more efficiently compared with DCs from other vaccinated mice. (A) Flurorescein isothiocyanate-dextran uptake by splenic DCs during the incubation period of 0–60 min. (B) DQ-OVA processing by splenic DCs during the incubation of 60 min. GM-CSF, granulocyte-macrophage colony-stimulating factor; DC, dendritic cells; CS, chitosan; OVA, ovalbumin; JE, Japanese encephalitis; pJME, premembrane and envelope proteins derived from JE virus; MFI, mean fluorsecence intensity.

To examine the ability of DCs to capture Ags, FITC-dextran uptake was monitored. Splenic DCs from the treated mice were incubated with 2 mg/ml FITC-dextran for 0–60 min to enable dextran internalization. The uptake of FITC-dextran was quantified by flow cytometry. The rates of Ag capture by splenic DCs by the chitosan-pJME-CSF nanoparticles and pJME/GM-CSF were higher compared with that by pJME and the rate of Ag capture by splenic DCs produced by chitosan-pJME/GM-CSF nanoparticles was higher compared with that by pJME/GM-CSF (Fig. 5A). Previous reports have demonstrated that FITC-dextran uptake by splenic DCs from pGM-CSF-treated mice was not saturable to 5 mg/ml and was not inhibited by cytochalasin D (18,19).

### Ag processing

The chitosan-pJME/GM-CSF nanoparticle and pGM-CSF-generated DCs internalized the majority of the DQ-OVA, as they are more efficient at capturing Ag. The rate of OVA processing within the first 30 min (slope of the line between 0 and 30 min) was 3-fold higher in the chitosan-pJME/GM-CSF nanoparticle-generated DCs compared with that in the pJME/GM-CSF-generated DCs (Fig. 5B).

### Capacity to stimulate T cell proliferation

**Figure 6:**
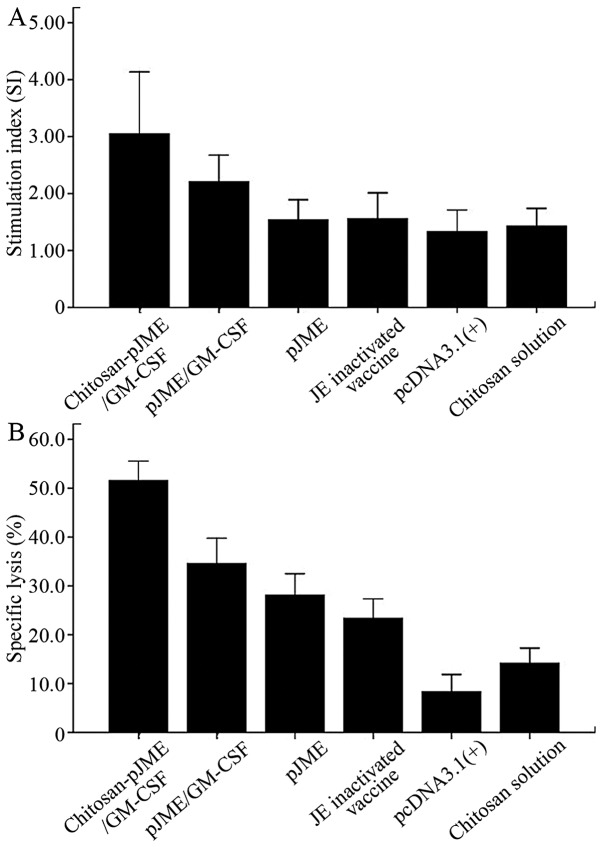
Lymphocyte proliferation and specific lysis of JEV-infected P815 cells by DNA-vaccinated spleen cells stimulated with JEV. (A) T cell immunity generated by DNA vaccines was assayed. Splenocytes collected 3 weeks after the final immunization were examined for JEV-specific pro-liferative responses. (B) Specific lysis of JEV-infected P815 cells by DNA vaccine immunized spleen cells stimulated with JEV. Cytotoxic activities against JEV-infected P815 cells were measured at an E:T ratio of 10:1 using LDH activity as a marker of cell lysis. Data is expressed as the mean percentage ± standard deviation, *n*=4 of specific lysis. GM-CSF, granulo-cyte-macrophage colony-stimulating factor; JEV, Japanese encephalitis vaccine; pJME, premembrane and envelope proteins derived from JE virus; LDH, lactate dehydrogenase.

The ability to stimulate the proliferation of T cells from DCs in the chitosan-pJME/GM-CSF nanoparticle- and pJME/GM-CSF-vaccinated groups were significantly enhanced (P<0.05) compared with the pJME and JE-inactivated vaccines, and this was signififcantly increased in the chitosan-pJME/GM-CSF nanoparticle group compared with the pJME/GM-CSF group (P<0.05; Fig. 6A).

### CTL activity

CTL activity assays were performed using an LDH activity release test. Following adjustment of the effecter-to-target cell ratio to 10:1, the CTL activities of the spleen cells from the Balb/c mice from the chitosan, pcDNA3.1(+), JE-inactivated, pJME-group, pJME/GM-CSF and chitosan-pJME/GM-CSF nanoparticle groups were 14.2, 8.36, 23.4, 28.1, 34.6 and 51.60%, respectively (Fig. 6B).

## Discussion

The use of plasmid DNA for immunization has emerged as a powerful approach to develop novel vaccines. In our previous studies, it was found that plasmid DNA immunization induced lower titers of JEV neutralizing antibodies compared with a commercial vaccine (33,34). This suggested that, to enhance the efficacy of the JEV DNA vaccine, novel methods require development to result in enhanced anti-JEV cellular and humoral immune responses. In the present study, chitosan was used to produce nanoparticles and to provide a positive charge on the particles for adsorption of the plasmid DNA.

Chitosan nanoparticles were obtained by coacervation between chitosan and pJME/GM-CSF. The size of the prepared nanoparticles, determined using a zetasizer was found to be 108.3 nm. Transmission electron microscopy also confirmed that the chitosan-pJME/GM-CSF nanoparticles were ~50–150 nm in size and almost spherical in shape. The gel retardation experiment demonstrated that pJME/GM-CSF was almost completely retarded in the loading well, suggesting that the majority of the pJME/GM-CSF binded with chitosan, which was in accordance to the result obtained from the measurement of entrapment efficiency (92%). The Zeta potential of the prepared nanoparticles was found to be 10.8±1.3 mV at the complexation pH (pH 5.5) and 2.9±0.6 mV at pH 7. The cationic character of chitosan is a crucial parameter for the formation of complexes between the polysaccharide and DNA. The p*K*a of the amino group in the repeating units is 6.5, rendering >90% of amino groups protonated at pH 5.5, while at a physiological pH, the majority of the positive charge is neutralized (35). This unique property ensures that nanoparticles, which form at a low pH remain physically stable at a physiological pH. The chitosan-pJME/GM-CSF nanoparticles were also evaluated for their ability to protect DNA against degradation by DNAse I. Chitosan effectively protected the pJME/GM-CSF from degradation in high concentrations of DNAse I. In addition, the concentration of nucleases *in vivo* was markedly lower compared with that *in vitro* and thus, this protection to DNA by chitosan may be important for the maintenance of integration and function of a DNA vaccine.

In order to investigate the local effects of plasmid-expressed GM-CSF in muscle, infiltration cells were observed by H&E staining and immunohistochemical methods. It was observed that pJME/GM-CSF recruited large and multifocal conglomerates of macrophages and granulocytes, the majority of which were neutrophils. During the peak of infiltration, an appreciable number of immature DCs appeared, although no T or B-cells were detected. This result differed from that of a study by McKay *et al* (36), which found that plasmid GM-CSF resulted in the recruitment of macrophages to the site of inoculation and specifically augmented vaccine-elicited CD4+ T lymphocyte responses. Haddad *et al* (32) did not detect the presence of DCs at the injection site following DNA vaccine immunization, while Oka *et al* (37) detected the presence of DCs following DNA vaccine immunization. These differences may be due to the properties of different plasmid-based vectors, properties unique to the Ag involved, different forms of the adjuvants, different time-periods of detecting cell infiltration, missing of infiltrated cells by the sectioning procedure, different Abs or other unknown biases.

It has been well-established that the *in vitro* culture of bone marrow or CD34+ peripheral blood mononuclear cells with GM-CSF and IL-4 leads to the outgrowth of immature DCs, which may develop into mature DCs following a variety of inflammatory stimuli (38,39). At the immune site, the recruitment by pJME/GM-CSF involves infiltration by multiple cells, including non-mature DCs. It is possible that following pJME/GM-CSF immunization, GM-CSF expressed *in vivo* may have similar effects on circulating DC precursors recruited into the injection site, with DC maturation resulting from the low grade inflammatory response observed following any DNA injection. At present, the mechanism underlying the recruitment of infiltrating cells remain to be elucidated.

The correlation between surface phenotype and the functional status of DCs has become a widely-accepted means of assessing DC maturation (40,41). Immature DCs in peripheral tissues are characterized by high Ag capture capacity, high intracellular MHC class II levels and low levels of expression of costimulatory molecules, including CD80 and CD86. Stimuli, including pathogens and inflammatory mediators cause DCs to mature and migrate to lymphoid tissues. Upon DC maturation, the Ag capturing activity is downregulated, the surface expression of costimulatory molecules is upregulated and class II molecules are translocated from intracellular compartments to the cell surface (40,41). In the present study, costimulatory molecules on splenic DCs from each group were detected. pJME/GM-CSF increased the expression of MHCII on the DCs, as well as the capacity for Ag capture, processing and presentation. This suggested that GM-CSF promoted the maturation of DCs and enhanced their function. Similar to the results of a study by Daro *et al* (19), the pJME/GM-CSF-generated DCs did not exhibit all the characteristics expected of either mature or immature DCs, and the reason for this remains to be elucidated. The expression of costimulatory molecules was not detected, which was possibly associated with poor antibody sensitivities.

In order to assess the cell-mediated response induced by the DNA vaccine, the Ag-specific CTL activity of mouse splenic cells was assessed. The CTL activity produced by pJME was significantly higher compared with that of the JE-inactivated, chitosan and pcDNA3.1(+) vaccines, suggesting that pJME induced a more marked cell-mediated response compared with the JE-inactivated vaccine. The CTL activity induced by pJME/GM-CSF was significantly higher compared with that of pJME. Therefore, pJME/GM-CSF induced more marked cell-mediated immunity compared with pJME.

The types and quantities of infiltrated cells recruited by the chitosan-pJME/GM-CSF nanoparticle group were similar to those of the pJME/GM-CSF group, although the peak of cell infiltration was of longer duration. The possible reason for this was that nanoparticles were gradually released in the tissues and, therefore, maintained a relatively stable level of DNA vaccine to continuously stimulate immunity. Compared with the pJME/GM-CSF group, the chitosan-pJME/GM-CSF nanoparticle group significantly increased the number of splenic DCs, the expression of MHCII on the splenic DCs, Ag capture and the processing and presentation functions of splenic DCs, and increased the cell-mediated immunity induced by the vaccine. Chitosan did not affect the number, phenotype or function of the splenic DCs, suggesting that chitosan enhanced the immunoadjuvant properties of GM-CSF. The possible reason for this was that the chitosan-pJME/GM-CSF nanoparticles protected the pJME/GM-CSF from degradation by nuclease, resulting in the increase in the expression level of prME/GM-CSF. However, direct evidence of the role of chitosan-pJME/GM-CSF nanoparticles requires further investigation for confirmation.

The significant increase in the levels of mouse spleen APCs in the chitosan-pJME/GM-CSF nanoparticle group may be associated with the aggregation ability of macrophages by chitosan (42). Overall, pJME/GM-CSF recruited numerous types of cells at the injection site, including immature DCs, increased the number of splenic DCs and the expression of MHCII on the splenic DCs, enhanced the Ag capture, processing and presentation functions of splenic DCs, and the cell-mediated immunity induced by the vaccine. This suggested that the immune-enhancing effects of pJME/GM-CSF were associated with the size and appearance of the infiltrate of CD11c+ cells. This provided experimental evidence for further investigation and application of GM-CSF. The intramuscular injection of chitosan-pJME/GM-CSF nanoparticles prolonged the duration of cell infiltration at the injection site, increased the number of DCs and improved the functions of the DCs. These results revealed that chitosan-pJME/GM-CSF nanoparticles enhanced the immunoadjuvant properties of GM-CSF via intramuscular injection and provides experimental evidence for the wider application of chitosan. In addition to its inherent safety, biodegradability and multifunctionality, chitosan offers a promising potential delivery platform for DNA vaccines.

## Figures and Tables

**Table I:** Immunohistochemical analysis of muscles from BALB/c mice 3 days after immunization.

Group	Infiltrate grade	Mac-3 I−A^d^/I−E^d^	CD11c	GR-1
Chitosan-pJME/GM-CSF	3.0+	3.0+	2.0+	2.0+
pJME/GM-CSF	3.0+	3.0+	1.75+	1.25+
pJME	1.5+	2.0+	0.75+	1.25+
JE-inactivated vaccine	1.0+	2.0+	0.75+	1.5+
pcDNA3.1(+)	0.75+	0.5+	0.25+	1.5+
Chitosan solution	0.25+	0.5+	0	1.25+

Data are presented as the mean ± standard deviation from four muscle sections. Infiltrate size was graded on hematoxylin and eosin sections, as described in Fig. 2 the Materials and methods. Cell markers were predominantly expressed by macrophages, dendritic cells and granulocytes. 0=no infiltrate; 1+=one small cell cluster; 2+=two small or moderate size cell clusters and 3+=extensive, multifocal cell infiltration. GM-CSF, granulocyte-macrophage colony-stimulating factor; JE, Japanese encephalitis; pJME, premembrane and envelope protein of JEV; CD11c; integrin, αx.
